# Developmental brain changes during puberty and associations with mental health problems

**DOI:** 10.1016/j.dcn.2023.101227

**Published:** 2023-03-09

**Authors:** Niousha Dehestani, Sarah Whittle, Nandita Vijayakumar, Timothy J. Silk

**Affiliations:** a School of Psychology, Deakin University, 221 Burwood Highway, Burwood, VIC 3125, Australia; b Melbourne Neuropsychiatry Centre, Department of Psychiatry, The University of Melbourne and Melbourne Health, VIC, Australia; c Developmental Imaging, Murdoch Children’s Research Institute, Parkville 3052, Australia; d Centre for Adolescent Health, Murdoch Children's Research Institute, Parkville, VIC, Australia

**Keywords:** Brain development, Mental health problems, Puberty, Pubertal timing, Brain age

## Abstract

**Background:**

Our understanding of the mechanisms relating pubertal timing to mental health problems via brain development remains rudimentary.

**Method:**

Longitudinal data was sourced from ∼11,500 children from the Adolescent Brain Cognitive Development (ABCD) Study (age 9–13years). We built models of “brain age” and “puberty age” as indices of brain and pubertal development. Residuals from these models were used to index individual differences in brain development and pubertal timing, respectively. Mixed-effects models were used to investigate associations between pubertal timing and regional and global brain development. Mediation models were used to investigate the indirect effect of pubertal timing on mental health problems via brain development.

**Results:**

Earlier pubertal timing was associated with accelerated brain development, particularly of subcortical and frontal regions in females and subcortical regions in males. While earlier pubertal timing was associated with elevated mental health problems in both sexes, brain age did not predict mental health problems, nor did it mediate associations between pubertal timing and mental health problems.

**Conclusion:**

This study highlights the importance of pubertal timing as a marker associated with brain maturation and mental health problems.

## 1 Introduction

Adolescence is characterized by significant biological, social, and emotional changes that establish a foundation for adulthood. Crucial biological milestones are triggered by the production of pubertal hormones, which lead to physical changes (i.e., gonadal development and secondary sexual characteristics such as breast and pubic hair changes) and ultimately support reproductive maturation (Sawyer et al., 2018). Adolescence is also accompanied by dynamic changes in social and emotional functioning. While most are able to navigate these changes successfully, it is also a period of increased vulnerability to mental health problems (Choudhury, 2010). Crucially, individual differences in pubertal maturation have been shown to influence both mental health problems and the onset of clinical-level disorders (Ullsperger and Nikolas, 2017). Pubertal changes have also been shown to influence the developing brain, which represents one potential mechanism that confers the development of mental health problems (Vijayakumar et al., 2018). Indeed, the brain undergoes significant structural changes during adolescence, and individual differences in the development of brain structure have been shown to predict the severity of mental health problems (Cropley et al., 2021, Kaufmann et al., 2019).

Although adolescents progress through the same stages of puberty, there is individual variability in the age of onset and the progression through pubertal stages relative to peers of the same sex. This variability in pubertal stage at any given age is referred to as ‘pubertal timing’ (Byrne et al., 2017). Previous studies have demonstrated that pubertal timing is associated with mental health problems and clinical-level disorders. However, there have been inconsistent findings regarding the nature and direction of these associations due to varied measurements of pubertal timing across studies (Mendle, 2014, Ullsperger and Nikolas, 2017). We recently established a new method for calculating pubertal timing called ‘puberty age’ (Dehestani et al., 2023, preprint; preprint: https://www.medrxiv.org/content/10.1101/2022.05.13.22275069v1), which addresses limitations in existing studies such as the use of limited features of puberty and a focus on linear patterns of development. Building upon the ‘brain age’ literature, our method uses multiple features of puberty, nonlinear modelling, and out of sample prediction to increase generalizability of findings. In this approach, a supervised machine learning model learns the relationship (accommodating both linear and nonlinear trends) between chronological age and multiple pubertal features (physical markers and hormonal levels) in a training sample, with the group-level relationship termed ‘puberty age’. The fitted model is tested in the new ‘test’ sample to form out-of-sample predictions. Further, by subtracting an individual’s chronological age from group-level normative ‘puberty age’, we identify their ‘puberty age gap’ that reflects pubertal timing for each individual relative to the group average. In this normative model, a positive puberty age gap indicates relatively earlier pubertal timing, and a negative puberty age gap indicates relatively delayed pubertal timing. We used this technique to investigate the association between pubertal timing and mental health problems, and findings indicated that a positive puberty age gap (i.e., earlier timing) was associated with greater symptoms across most psychopathology domains in males and females during early adolescence (Dehestani et al., 2023, preprint). These findings are aligned with the “*maturation disparity*” and “*developmental readiness”* hypotheses, which suggest that the mismatch between physical development and progression of emotional and cognitive development may increase in those with early pubertal timing and result in the development of mental health problems (Brooks-Gunn et al., 1985, Petersen et al., 1988).

One of the mechanisms by which early pubertal timing might contribute to mental health problems is via its impact on the developing brain. Numerous studies have highlighted the importance of individual differences in structural brain maturation for explaining risk for mental health problems (Mattoni et al., 2021, Merz et al., 2018, Schmaal et al., 2016, Schmaal et al., 2017). While few studies have directly sought to examine the relationship between pubertal timing and brain structure, a number of studies have begun to tap into these associations through statistical modelling approaches (Vijayakumar et al., 2018). Specifically, by controlling for chronological age when examining the association between pubertal stage and brain structure, previous research has shown that greater stage-for-a-given-age is related to smaller global grey matter volume (Bramen et al., 2011). However, a number of null associations have also been noted in both females and males (Koolschijn et al., 2014; Peper et al., 2009a). At a regional level, most existing studies have focused on the prefrontal cortex, generally reporting that greater stage-for-a-given-age is associated with smaller structure or thinner cortices (in particular the orbitofrontal and anterior cingulate cortices (Peper et al., 2009b; Koolschijn et al., 2014)). Associations between pubertal stage and subcortical volumes (controlling for age) have also been commonly investigated, although results are largely inconsistent, and sometimes in opposite directions for males and females (Blanton et al., 2012, Hu et al., 2013, Urošević et al., 2014).

Of note, pubertal stage only captures variation in secondary sexual characteristics and cannot capture the initial stages of puberty, when underlying hormonal changes are not yet associated with observable physical changes (Dorn and Biro, 2011). Some studies have investigated the effect of hormone levels on the brain, while similarly accounting for confounding effects of age. For example, studies have reported that higher levels of hormones-for-a-given-age have a significant negative association in females and positive association in males with cortical thickness, particularly in prefrontal regions (Bramen et al., 2011). Nevertheless, other studies observed no such associations (Koolschijn et al., 2014). Additionally, in terms of subcortical regions, levels of hormones-for-a-given age have been associated with amygdala but not striatal volume (Barth et al., 2015, Brouwer et al., 2015, Neufang et al., 2009, Peper et al., 2009). Overall, most existing studies have focused on a limited number of regions, most commonly the prefrontal cortex and subcortex. Given that pubertal hormones are known to affect the functioning of a wider set of brain regions (Byrne et al., 2017), there is value in assessing associations between differing features of pubertal timing and the development of regions across the whole brain.

Changes in brain structure during adolescence have also been shown to relate to mental health problems (Mueller et al., 2013, Sharma et al., 2013). As mentioned above, ‘brain age’ has gained recent attention as a method to examine individual differences in global brain maturity. As with our ‘puberty age’ approach, ‘brain age’ is calculated by predicting chronological age from multiple brain features measured through neuroimaging (such as regional cortical volumes) across individuals, and subtracting each individual’s chronological age from group-level ‘brain age’ captures their ‘brain age gap’ (Cole and Franke, 2017). A positive brain age gap is thought to reflect a relatively older brain for a given age, while a negative brain age gap reflects a relatively younger brain. A number of studies have examined the relationship between brain age gap and mental health problems, with lifespan or adult studies identifying significant associations between brain age gap and psychosis, anxiety, and major depressive disorders (Franke and Gaser, 2019). While studies are limited and findings mixed in developmental samples, there is some indication that positive brain age gap, or more mature brain, during adolescence (most likely reflecting a reduction in grey matter size within the context of normative patterns of adolescent brain development) may also be significantly associated with certain mental health problems (Cropley et al., 2021). Moreover, both pubertal timing and brain age are considered markers of biological aging and may represent similar pathways to adolescent-onset mental health problems (Colich et al., 2019). Therefore, investigating the association between pubertal timing and brain age gap may help us better understand the biological underpinnings of mental health problems.

The aim of this study was to examine the relationships between pubertal timing (i.e., puberty age gap), brain development (i.e., brain age gap) and mental health problems. Brain age analyses were conducted at two levels; first at a coarse whole-brain level, and then, further teasing apart the influence of different lobes/regions. Based upon prior literature that has identified reductions in grey matter size during puberty, it was hypothesized that earlier pubertal timing (i.e., positive puberty age gap) would be associated with a more mature brain (i.e., positive brain age gap). We also investigated whether brain age gap mediates the relationship between puberty age gap and mental health problems. We have previously reported positive associations between puberty age gap and multiple dimensions of psychopathology (Dehestani et al., 2023, preprint) and expected this relationship to be mediated via positive brain age gap.

## 2 Method

The sample was derived from the Adolescent Brain Cognitive Development (ABCD) Study, a large population-representative cohort in the United States of America. This is an ongoing project that longitudinally follows ∼11,500 children (47% females, 9 or 10 years at baseline) through annual assessment waves. Refer to Garavan et al. (2018) for further details on the recruitment protocols. We aimed to study brain maturation using a normative modelling approach. We used data from release 4.0 of ABCD, which included pubertal and mental health measures as well as neuroimaging data from baseline and two-year follow-up. In order to capture the widest possible age range, but to avoid the potential bias created from longitudinally repeated measures in the sample, we randomly selected a single time point from every individual. Additionally, we removed participants who had missing data for measures of puberty, mental health problems, demographic information, or grey matter structure. From the remaining sample, we only included participants for whom the imaging data was recommended by ABCD (abcd_imgincl01, see more details in Supplementary Information [SI], Section 1). Further details about the data cleaning process for the sample used in the current study (such as removing missing values and cleaning hormone data) are found in SI, Section 1. The final sample size for this study was N = 10,167 participants (N = 4894 females) with an age range of 9–13. Some analyses only used a subset of this sample, which is outlined in detail below. We also utilized the Human Connectome Project Development (HCP-D) sample for supplemental analyses (SI, Section 7), which includes 652 participants with the age range of 8–22 years.

### 2.1 Measures

#### 2.1.1 Grey matter structure

T1-weighted images were acquired using 3 T MRI scanners (Phillips, Siemens, or General Electric [GE]). For further details on the imaging protocol and the acquisition, refer to Casey et al. (2018). Data processing was conducted by the ABCD Data Analysis and Informatics Core. Images were processed through Free Surfer 5.3.0 to extract regional estimates of structure. The current study used cortical regional estimates of volume, thickness and surface area based on the Desikan-Killiany parcellation (Alexander et al., 2019) and default subcortical segmentation. Further, data was restricted to those recommended by ABCD study quality checks (see SI, Section 1).

#### 2.1.2 Hormones

Participants provided saliva samples on the day of their MRI assessment, which were collected via passive drool. They were assayed in duplicate for pubertal hormone levels using Salimetrics enzyme-linked immunosorbent assay (ELISA) kits. The current study focused on testosterone (T) and dehydroepiandrosterone (DHEA) levels. Although estradiol levels were also measured in females, there were missing values for approximately 780 participants and thus excluded from the analyses. Refer to the SI for further detail on processing of hormone data (e.g., accounting for wake-up time, exercising, or consuming food and caffeine prior to saliva collection) and calculation of a single hormone value from duplicate assays (according to protocols outlined by Herting et al. (2021).

#### 2.1.3 Pubertal developmental scale

The Pubertal Developmental Scale (PDS; Petersen et al., 1988) was used to assess physical changes during puberty, including growth spurt, body hair and skin changes for both females and males, breast development and menarche for females, and facial hair and voice deepening for males. Items were rated on a Likert scale of 1–4, from “has not begun” to “seems complete”. The current study utilized parent-report of the PDS as it may be more reliable compared to self-report for the age range of our sample (Rasmussen et al., 2015).

#### 2.1.4 Mental health problems

The parent-reported Child Behaviour Checklist (CBCL; Achenbach and Ruffle, 2000) was used to examine mental health problems. The current study focused on the externalizing and internalizing problem subscales, as well as the overall “total problems” scale. Moreover, we repeated analyses with syndrome scales for withdrawn/depressed, somatic complaints, anxious/depressed, social problems, thought problems, attention problems, rule-breaking behavior and aggressive behavior as outcome variables.

#### 2.1.5 Body mass index

Weight and height were assessed at each wave by researchers, with an average height and weight calculated from multiple measurements. Using this data, we calculated Body Mass Index (BMI) according to the CDC 2000 Growth Charts (Gray et al., 2020).

#### 2.1.6 Socioeconomic status (SES)

Socioeconomic status was assessed for each family based on highest level of education and mean income of parents (self-reported by parents).

#### 2.1.7 Race/ethnicity

Race and Ethnicity, divided into five categories: White, Black, Hispanic, Asian, and Other/Multi-race, was reported by parents.

### 2.2 Statistical analyses

We first generated the “brain age” and “puberty age” models, using a randomly selected single timepoint for each participant from baseline and two-year follow-up waves (as the extended age range captures more variance across development and supports improved accuracy of the model; de Lange et al., 2022). Next, we examined associations between brain age gap and puberty age gap in this dataset. Subsequently, using brain age gap and puberty age gap at baseline, mediation models were conducted predicting mental health problems at two-year follow-up.

#### 2.2.1 Brain age

Brain age was computed by using Support Vector Regression (SVR) with a radial basis function (RBF) kernel as a nonlinear supervised machine learning approach to predict chronological age from brain features including regional cortical thickness, volume, and surface area, as well as subcortical volumes. To reduce the number of input model features and avoid potential overfitting, we first employed Principal Component Analysis (PCA) to identify the first N principal components that explained the majority (90%) of individual variations in regional brain features (i.e., orthogonal dimensions of brain structure). The selected N = 90 components captured 90% of the total variance of all input measures (see SI Section 2). Next, the SVR model learned the relationship between the principal brain components and chronological age. This fitted model was then used to predict chronological age from brain components in an independent test sample. The regression to mean (RTM) bias-adjusted predictions reflected “brain age” and subtracting chronological age from brain age yielded “brain age gap” for each participant. Positive and negative brain age gaps suggest, respectively, an older and younger brain relative to an individual’s chronological age. two-year follow-up from each participant (N = 10,167 participants (N = 4894 females)) was randomly selected and used to implement the brain age model. Crucially, model training was performed on a “typically developing” group, based on scores in non-clinical ranges across the CBCL DSM-5 oriented scales (depression, anxiety, somatic, attention-deficit-hyperactivity, oppositional defiant, conduct, obsessive-compulsive, sluggish cognitive tempo, and stress) (N = 5145 participants (2582 females)). Refer to SI, Section 1 for more details. A nested 10-fold cross-validation design such that in every fold, 90% of the sample was used for training, and the remaining 10% of the sample was used for testing as validation process of model in the typically developing sample. A nested inner loop (10-folds) was used for SVR hyperparameter tuning. A group shuffle split was used across the folds with family ID as the group indicator to ensure that no two related individuals were split across train and test sets. This ensures that siblings belong either to the train or test set and alleviates any potential impacts of familial information on model predictions. Finally, we applied the fitted model to the whole sample (i.e., beyond the “typically developing” group) to examine the associations with puberty age gap and mental health problems.

Supplemental analyses repeated the brain age model using data from Human Connectome Project Development (HCP-D, N = 622 participants) that covers a wider age range of 8–22 years old. We implemented our brain age model on HCP as our training sample and tested the fitted model on ABCD. This was undertaken to ensure that the narrow age range of the ABCD cohort did not obscure any association between brain age gap and our variables of interest. For more details, refer to SI, Sections 7.

#### 2.2.2 Puberty age

We calculated pubertal timing (for males and females separately) using the new ‘puberty age’ method proposed in our recent study (Dehestani et al., 2023, preprint). As discussed above, the method applied the concepts from the brain age literature to predict chronological age from pubertal features, including hormones and physical changes. Subtracting chronological age from RTM bias-adjusted puberty age is referred to as “puberty age gap”. Positive and negative puberty age gaps reflect early and late pubertal timing for an individual’s chronological age, respectively. Briefly, we used a generalized additive model (GAM) within a nested 10-fold cross-validation (trained in 90% of the sample and tested in 10% of the remaining sample). Inner loop hyperparameter tuning was performed by a grid search for optimal regularization penalty on each term. We implemented three different methods for calculating puberty age gap: i) hormonal puberty age based on hormone levels alone, ii) physical puberty age based on PDS items alone, and iii) combined puberty age that incorporates both hormonal and physical information. To implement puberty age models, we used data from baseline and two-year follow-up and we randomly selected a single time point for each participant (N = 10,167 participants (N = 4894 females with each family restricted to either the train or test sample. Additionally, we trained our puberty age model on a “typically developing” group (i.e., those with scores < 60 on the CBCL DSM-5 oriented scales; (N = 5145 participants (2582 females)). For further details, see (Dehestani et al., 2023, preprint). Finally, we applied the fitted model to the whole sample (i.e., beyond the “typically developing” group) to examine the associations with brain age gap and mental health problems.

#### 2.2.3 Associations between puberty age gap and brain age gap

Linear mixed effect (LME) models were used to examine associations between puberty age gap and brain age gap cross-sectionally at baseline. We randomly sampled one participant from each family ID so as not to capture familial/genetic effects. We examined whether puberty age gap predicted brain age gap (using Formula 1). Age was modelled as a fixed effect and site as a random effect. Models were examined separately in males and females and repeated for each of the three versions of puberty age gap (i.e., hormonal, physical, and combined puberty age gap). Correction for multiple comparisons (across the three puberty age gap models) was undertaken at False Discovery Rate (FDR) of 0.05. Supplemental models included the covariates of BMI, race and SES (see details in SI, Section 4).

(1) Brain age gap ∼ puberty age gap + Age + (1|multisite)

Although the brain age method narrows the complexity of brain features, it might obscure important regional patterns of association with puberty. To evaluate the contribution of specific lobes and regions, we used a leave-one-lobe/region-out (LOLO or LORO) design (as used in previous brain age literature (Rakesh et al., 2021). We first focused on lobar contributions and re-trained the brain age model 6 times in a LOLO design that removed one specific lobe in each iteration (subcortex, insular, temporal, parietal, occipital, and frontal lobes). Next, we implemented the same LME models (Formula 1) to examine associations between each LOLO brain age gap and puberty age gap. Subtracting the previous T-statistic of LME associations between (whole) brain age gap with puberty age gap from the T-statistic yielded from LOLO brain age gap indexes the relative contribution of each lobe to the global association. A greater T-statistic reduction reflects stronger contributions of a given lobe. This analysis was also repeated in a LORO design by removing each single brain feature (i.e., a specific cortical (cortical volume, thickness and area for each region) or subcortical region (subcortical volume)) to capture the contribution of each single region in the association between puberty age gap and (whole) brain age gap.

#### 2.2.4 Mediating role of brain age gap in the association between puberty age gap and mental health problems

**Fig. 1:**
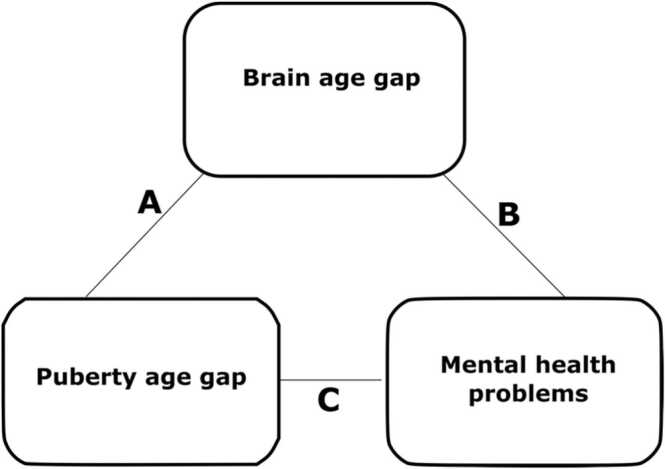
Visualisation of the mediation of puberty age gap to mental health problems via brain age gap A: brain age gap ∼ puberty age gap + age + (1| site). B: mental health problems ∼ puberty age gap + brain age gap + age + baseline mental health problems + (1| site). C: mental health problems ∼ puberty age gap + baseline mental health problems + age + (1| site).

We examined whether brain age gap (at baseline) mediated the relationship between puberty age gap (baseline) and mental health problems at two-year follow-up (see Fig. 1). The sample size for this analysis was N = 4138 participants (1970 female). Mediation was tested using bootstrapped confidence intervals (based on 1000 bootstrapped samples), calculated with the “mediation” package in R (Tingley et al., 2014). Again, we used LME models that modelled age and baseline mental health problems as fixed effects and multisite as a random effect. The mediation model was comprised of the following paths:

C represents the direct effect. The mediation (or indirect) effect is calculated as A*B> 0. This analysis also controlled for mental health problems at baseline. In post-hoc analyses, we examined mediation via lobar brain age. Based on our LOLO results, this was limited to subcortical and frontal lobes in females and the subcortical lobe in males. Thus, we implemented a subcortical brain age model by only using subcortical regions as input features to predict age for females and males; similarly, frontal brain age was derived from features of regions in the frontal lobe for females. Next, chronological age was subtracted from lobar brain age to calculate lobar brain age gap to use in the mediation model explained above. This analysis was also repeated with the inclusion of BMI, SES and race as a covariate (SI, Sections 5 and 6).

## 3 Results

### 3.1 Accuracy of brain age and puberty age models

**Fig. 2:**
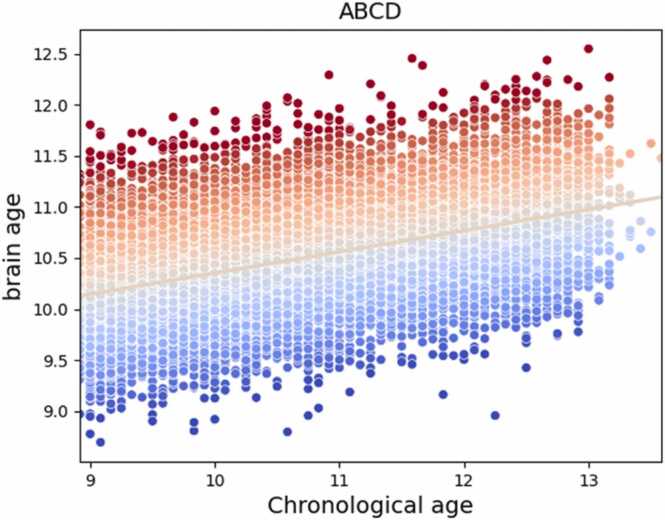
Predicted brain age plotted against chronological age. The colors on the scatter plot reflect brain age gap with red indicating a positive brain age gap and blue indicating a negative brain age gap.

There was adequate out-of-sample accuracy over 10-fold cross-validation of the brain age model (r = 0.47 p < 0.001, MAE = 7.5 months, see Fig. 2), and similarly the combined puberty age model (r = 0.64, p < 0.001, MAE = 7.80 months [females]; r = 0.60, p < 0.001, MAE = 8.20 months [males]), physical puberty age model (r = 0.61, p < 0.001, MAE = 8.02 months [females]; r = 0.50, p < 0.001, MAE = 8.90 months [males]), and hormonal puberty age model (r = 0.42, p < 0.001, MAE = 9.04 months [females]; r = 0.51, p < 0.001, MAE = 8.80 months [males]).

### 3.2 Examining the association between different models of puberty age gap and brain age gap

**Fig. 3:**
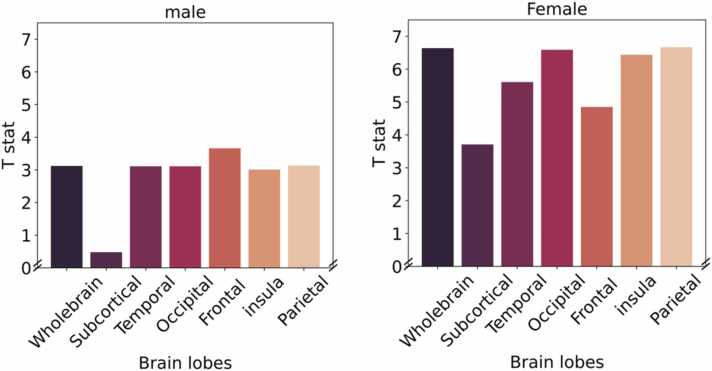
Contribution of each lobe in the association between physical puberty age gap and brain age gap, examined using the leave-one-lobe-out approach. Greater reduction in the T-statistic for a given lobe (relative to the “global” brain age) suggests a stronger contribution of this lobe.

**Fig. 4:**
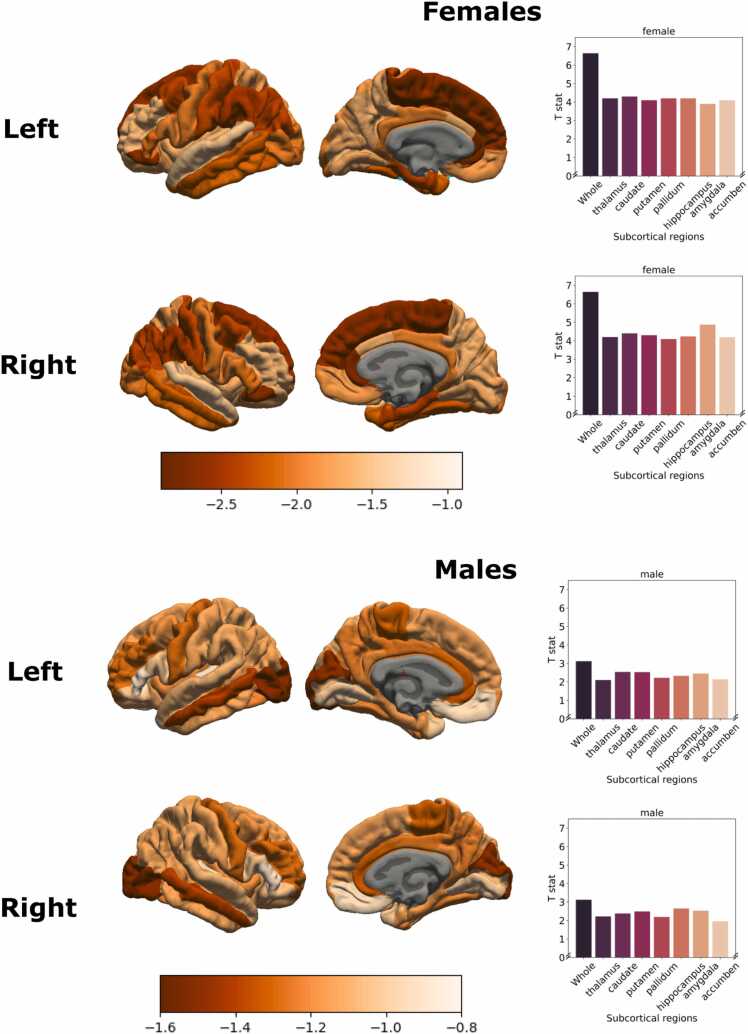
Contribution of each cortical region and each region of subcortical in both left and right hemispheres in the association between puberty age gap and brain age gap, examined using the leave-one-region-out approach. Greater reduction in the T-statistic for a given region (relative to the “global” brain age) suggests a stronger contribution of this lobe.

Analyses revealed a significant association between puberty age gap and brain age gap, such that positive puberty age gap was associated with positive brain age gap. All three measures of puberty age gap were related to brain age gap, but the strongest association was identified for physical puberty age gap (in both males and females, see Table 1). Post-hoc analyses interrogated regional contributions to the significant association between physical puberty age gap and brain age gap. In females, the greatest reduction in effect size (i.e., T-statistic) was observed for the subcortical and frontal lobes, and in males the greatest reduction in effect size was observed for the subcortical lobe, indicating the largest contribution to associations between physical puberty age gap and global brain age (see Fig. 3). The contribution of individual brain regions is presented in Fig. 4.

**Table 1:** Association between puberty age gaps and brain age gap.

	Female			Male		
	T	*p*	AIC	T	*p*	AIC
Physical puberty age	**6.64**	**< 0.001**	**26,281.38**	**3.12**	**< 0.001**	**28,977.04**
Combined puberty age	6.51	< 0.001	26,288.83	2.18	< 0.001	28,983.89
Hormonal puberty age	3.68	< 0.001	26,313.10	0.86	> 0.05	28,990.23

All *p*-values are FDR corrected

### 3.3 Mediation of the association between puberty age gap and mental health problems via brain age gap

Given that physical puberty age gap was most strongly associated with brain age gap, mediation models focused on this index of puberty age. Analyses failed to identify any significant mediation of the association between physical puberty age gap and mental health problems via brain age gap, when controlling for baseline mental health problems (see Table 2). Supplemental analyses examined these associations without controlling for baseline mental health problems (as there was little change in mental health problems between baseline and two-year follow-up). However these analyses also failed to identify significant associations between brain age gap and future mental health problems (see Supplementary, Section 5, Table S2). Finally, post-hoc analyses failed to identify significant mediation via subcortical and frontal brain age in females and subcortical brain age in males (see Supplementary, Sections 5 and 6, Table S3,4,5 and 6).

**Table 2:** Mediating role of brain age gap in the association between physical puberty age gap and mental health problems.

	Females			Males		
	ACME	ADE	Total effect	ACME	ADE	Total effect
Total problem	0.91	**< 0.01**	**< 0.01**	0.81	0.99	0.09
Externalising problem	0.45	**< 0.01**	**< 0.01**	0.71	0.71	0.20
Internalising Problem	0.84	**< 0.01**	**< 0.01**	0.60	0.62	0.20
Withdrawn/Depression	0.09	0.71	0.06	0.76	0.96	0.56
Anxiety/Depression	0.68	0.08	0.08	0.38	0.70	0.42
Somatic Complaint	0.81	**< 0.01**	**< 0.01**	0.78	0.59	0.11
Attention problems	0.45	0.61	0.55	0.34	0.77	0.56
Rule breaking	0.46	**< 0.01**	**< 0.01**	0.79	0.56	0.08
Aggressive behaviours	0.09	**< 0.01**	**< 0.01**	0.54	096	0.59
Thought problems	0.78	0.39	0.36	0.75	0.92	0.27
Social problems	0.73	**< 0.01**	**< 0.01**	0.68	0.68	0.09

All p values reported after FDR correction.

ACME = Average Causal Mediation Effect, ADE = Average Direct Effect

## 4 Discussion

The current study presents a novel investigation of the association between pubertal timing, brain development and mental health. Overall, our findings indicated positive associations between puberty age gap and brain age gap, suggesting that earlier pubertal timing is related to an older or more mature brain. While different methods of measuring pubertal timing (i.e., physical, hormonal, and combined multimethod measures) were all significantly related to brain structure, the strongest association was identified for physical puberty age gap in both males and females. Further, the subcortex exhibited the greatest contribution to associations between puberty age gap and (global) brain age gap in females and males, as well as the frontal lobe in females only. However, brain age gap was not associated with mental health problems, and nor was it found to mediate the association between puberty age gap and mental health problems.

As hypothesized, we identified positive associations between puberty age gap and brain age gap, suggesting more mature brain structure in those undergoing earlier pubertal timing. While prior literature has not directly examined the association between pubertal timing and brain age gap, they have reported negative associations between pubertal stage-for-age and (global and region) brain structure (Blanton et al., 2012, Peper et al., 2009). In other words, earlier pubertal maturation has been related to smaller/thinner cortices, given that the normative pattern of brain development is typically reflected by decreases in grey matter metrics across adolescence (Mills et al., 2016). While most of the literature has examined associations within frontal and subcortical regions, some studies have noted associations between pubertal stage-for-age and structure within occipital, temporal and parietal lobes (Vijayakumar et al., 2018). Our findings may thus reflect diffuse effects in the literature, as our brain age model summarizes global maturity across all brain regions. Furthermore, prior studies have typically investigated pubertal associations with specific morphological measurements such as cortical thickness or surface area, which have been shown to be (genetically) independent to one another (Panizzon et al., 2009). Our brain age model was also able to capture maturity across these metrics and can therefore facilitate interpretation about global patterns of advanced brain development in those undergoing puberty earlier than their peers.

Our findings revealed that physical puberty age gap had the strongest association with brain age gap, relative to pubertal timing models built on hormone measures or combined physical and hormonal pubertal features. Indeed, previous studies have also reported different patterns of association between brain structure and pubertal hormones vs. physical development (Peper et al., 2009b; Koolschijn et al., 2014, Bramen et al., 2011, Bramen et al., 2012). This may be because physical measures of puberty capture the cumulative effect of pubertal maturation, including observable manifestations of hormonal processes to date. Brain structure may be more likely to reflect these cumulative changes than a snapshot of hormone levels on a given day. As discussed above, hormone levels are also markedly influenced by momentary, non-pubertal factors, which may further confound associations with brain structure (Shirtcliff et al., 2009). Physical measures may also potentially capture psychosocial processes, reflecting social and cultural responses and changes to children entering puberty (Dorn et al., 2003, Dorn et al., 2006, Mendle, 2014), which are also known to influence brain structure (McLaughlin et al., 2019). Interestingly, no significant association was found between pubertal timing calculated from hormones and brain maturity in males, suggesting that psychosocial changes may have a stronger effect on brain structure, at least in the age range studied.

Furthermore, we found that associations between pubertal timing and global brain age were largely driven by subcortical and frontal lobes in females and subcortical regions in males. The role of the subcortex is consistent with animal research that highlights the influence of pubertal hormones on morphology of the amygdala, hippocampus and striatum, due to the prevalence of hormones receptors in these regions and accompanying changes in receptor density during puberty (Vijayakumar et al., 2018, Schulz et al., 2009, Sisk and Foster, 2004). A considerable literature in humans has also noted relationships between pubertal maturation and subcortical volumes in both males and females (Vijayakumar et al., 2021, Wierenga et al., 2018). The relative importance of the frontal lobe for females, but not males, may reflect earlier maturation of the prefrontal cortex in females during adolescence (Koolschijn and Crone, 2013, Lenroot and Giedd, 2010), and it is possible this also relates to earlier pubertal maturation in females relative to males during late childhood and early adolescence (Byrne et al., 2017). Therefore, an important future direction would be to assess these regional contributions at a later age in males. Nonetheless, overall these findings broadly support the role of puberty in the maturation of regions that support high-order cognitive and socioemotional processes.

However, we interestingly failed to identify significant associations between brain age gap and mental health problems, or significant mediation of the association between puberty age gap and mental health problems via brain age gap. Thus, although puberty age gap and brain age gap are associated, and we have previously reported associations between puberty age gap and mental health problems in this cohort (Dehestani et al., 2023, preprint), it appears that global brain age may not be sensitive to the neurobiological changes that mediate associations between pubertal timing and mental health problems. While unexpected, prior studies have similarly failed to identify associations between brain age gap and internalizing (Cropley et al., 2021, Rakesh et al., 2021) and externalizing problems (Cropley et al., 2021, Kaufmann et al., 2019). Even our measure of regional (lobar) brain age gap was not sensitive to any mediation effects, suggesting that perhaps traditional measures of regional brain structures with greater specificity (i.e., thickness / surface area of specific regions) may represent stronger markers of mental health problems. Indeed, other processes that may confer vulnerability for mental health problems during puberty, such as social and environmental risk factors, are known to have regional effects on the brain (McLaughlin et al., 2019) suggesting that greater specificity may be required to identify neural markers of mental health problems. Future research could therefore consider extending our analyses to examine the contribution of specific brain regions. It may also be fruitful to examine other features of brain maturity, such as white matter and functional connectivity.

The current study has its strength in the use of a large, openly available dataset, enabling assessment and replication with high statistical power. Nevertheless, this study has certain limitations to consider. Although we used parent-report of pubertal development, given evidence for validity in this age range, it would be valuable for future studies to replicate these results with clinician and/or self-report assessments. Secondly, as the cohort is a community sample, the majority of participants exhibited null or very few mental health problems. It would be important for future studies to test the efficacy of puberty and brain age in predicting more severe cases of mental health problems in clinical samples. Moreover, we did not control for menarche or phase of the menstrual cycle. Most of the sample however, had not undergone menarche, and menstrual cycle phase information (for those cycling females) was not available. Future research should consider the role of these factors in linking pubertal timing with brain development and mental health. Although the ABCD study is a unique dataset that has a large sample size with relatively rich pubertal information, the age range is restricted which may impact the accuracy of normative modelling. Finally, our analyses were restricted to late childhood / early adolescence, and further associations between brain age gap, puberty age gap and mental health problems may be evident in later adolescence.

In conclusion, this study highlighted earlier brain maturity in adolescents undergoing puberty earlier than their peers. We found that physical relative to hormonal measures of puberty had stronger associations with such global patterns of brain maturity. However, these global associations did not mediate the relationship between pubertal timing and mental health problems, suggesting that greater specificity in brain structure may be required to identify markers of mental health.

## Declaration of Competing Interest

The authors declare that they have no known competing financial interests or personal relationships that could have appeared to influence the work reported in this paper.

## Data Availability

I have shared the link to my data in the manuscript.

## References

[bib1] Achenbach T.M., Ruffle T.M. (2000). The child behavior checklist and related forms for assessing behavioral/emotional problems and competencies. Pediatr. Rev..

[bib2] Alexander B., Loh W.Y., Matthews L.G., Murray A.L., Adamson C., Beare R., Chen J., Kelly C.E., Anderson P.J., Doyle L.W., Spittle A.J., Cheong J.L.Y., Seal M.L., Thompson D.K. (2019). Desikan-Killiany-Tourville Atlas compatible version of m-CRIB neonatal parcellated whole brain atlas: The m-Crib 2.0. Front. Neurosci..

[bib3] Barth C., Villringer A., Sacher J. (2015). Sex hormones affect neurotransmitters and shape the adult female brain during hormonal transition periods. Front. Neurosci..

[bib4] Blanton R.E., Cooney R.E., Joormann J., Eugène F., Glover G.H., Gotlib I.H. (2012). Pubertal stage and brain anatomy in girls. Neuroscience.

[bib5] Bramen J.E., Hranilovich J.A., Dahl R.E., Forbes E.E., Chen J., Toga A.W., Dinov I.D., Worthman C.M., Sowell E.R. (2011). Puberty influences medial temporal lobe and cortical gray matter maturation differently in boys than girls matched for sexual maturity. Cereb. Cortex.

[bib6] Bramen J.E., Hranilovich J.A., Dahl R.E., Chen J., Rosso C., Forbes E.E., Dinov I.D., Worthman C.M., Sowell E.R. (2012). Sex matters during adolescence: Testosterone-related cortical thickness maturation differs between boys and girls. PLoS One.

[bib7] Brooks-Gunn J., Petersen A.C., Eichorn D. (1985). The study of maturational timing effects in adolescence. J. Youth Adolesc..

[bib8] Brouwer R.M., Koenis M.M.G., Schnack H.G., van Baal G.C., van Soelen I.L.C., Boomsma D.I., Hulshoff Pol H.E. (2015). Longitudinal development of hormone levels and grey matter density in 9 and 12-year-old twins. Behav. Genet..

[bib9] Byrne M.L., Whittle S., Vijayakumar N., Dennison M., Simmons J.G., Allen N.B. (2017). A systematic review of adrenarche as a sensitive period in neurobiological development and mental health. Dev. Cogn. Neurosci..

[bib10] Casey B.J., Cannonier T., Conley M.I., Cohen A.O., Barch D.M., Heitzeg M.M., Soules M.E., Teslovich T., Dellarco D.V., Garavan H., Orr C.A., Wager T.D., Banich M.T., Speer N.K., Sutherland M.T., Riedel M.C., Dick A.S., Bjork J.M., Thomas K.M., Dale A.M. (2018). The Adolescent Brain Cognitive Development (ABCD) study: Imaging acquisition across 21 sites. Dev. Cogn. Neurosci..

[bib11] Choudhury S. (2010). Culturing the adolescent brain: what can neuroscience learn from anthropology?. Soc. Cogn. Affect. Neurosci..

[bib12] Cole J.H., Franke K. (2017). Predicting age using neuroimaging: innovative brain ageing biomarkers. Trends Neurosci..

[bib13] Colich, N.L., Rosen, M.L., Williams, E.S., & McLaughlin, K.A. (2019). Biological Aging in Childhood and Adolescence Following Experiences of Threat and Deprivation: A Systematic Review and Meta-Analysis Dept. of Psychology, University of Washington, Seattle, WA, 2 Dept. of Psychology, Stanford Corresponding author: BioRxiv, 2(999), 1–85.

[bib14] Cropley V.L., Tian Y., Fernando K., Mansour L., S, Pantelis C., Cocchi L., Zalesky A. (2021). Brain-predicted age associates with psychopathology dimensions in youths. Biol. Psychiatry Cogn. Neurosci. Neuroimag..

[bib15] Dorn L.D., Biro F.M. (2011). Puberty and its measurement: A decade in review. J. Res. Adolesc..

[bib16] Dorn L.D., Dahl R.E., Williamson D.E., Birmaher B., Axelson D., Perel J., Stull S.D., Ryan N.D. (2003). Developmental markers in adolescence: implications for studies of pubertal processes. J. Youth Adolesc..

[bib17] Dorn L.D., Dahl R.E., Woodward H.R., Biro F. (2006). Defining the boundaries of early adolescence: A user’s guide to assessing pubertal status and pubertal timing in research with adolescents. Appl. Dev. Sci..

[bib18] Franke K., Gaser C. (2019). Ten years of brainage as a neuroimaging biomarker of brain aging: What insights have we gained?. Front. Neurol..

[bib19] Garavan H., Bartsch H., Conway K., Decastro A., Goldstein R.Z., Heeringa S., Jernigan T., Potter A., Thompson W., Zahs D. (2018). Recruiting the ABCD sample: Design considerations and procedures. Dev. Cogn. Neurosci..

[bib20] Gray J.C., Schvey N.A., Tanofsky-Kraff M. (2020). Demographic, psychological, behavioral, and cognitive correlates of BMI in youth: Findings from the Adolescent Brain Cognitive Development(ABCD) study. Psychol. Med..

[bib21] Herting M.M., Uban K.A., Gonzalez M.R., Baker F.C., Kan E.C., Thompson W.K., Granger D.A., Albaugh M.D., Anokhin A.P., Bagot K.S., Banich M.T., Barch D.M., Baskin-Sommers A., Breslin F.J., Casey B.J., Chaarani B., Chang L., Clark D.B., Cloak C.C., Sowell E.R. (2021). Correspondence between perceived pubertal development and hormone levels in 9-10 year-olds from the adolescent brain cognitive development study. Front. Endocrinol..

[bib22] Hu S., Pruessner J.C., Coupé P., Collins D.L. (2013). Volumetric analysis of medial temporal lobe structures in brain development from childhood to adolescence. NeuroImage.

[bib23] Kaufmann T., van der Meer D., Doan N.T., Schwarz E., Lund M.J., Agartz I., Alnæs D., Barch D.M., Baur-Streubel R., Bertolino A., Bettella F., Beyer M.K., Bøen E., Borgwardt S., Brandt C.L., Buitelaar J., Celius E.G., Cervenka S., Conzelmann A., Westlye L.T. (2019). Common brain disorders are associated with heritable patterns of apparent aging of the brain. Nat. Neurosci..

[bib24] Koolschijn P.C.M.P., Crone E.A. (2013). Sex differences and structural brain maturation from childhood to early adulthood. Dev. Cogn. Neurosci..

[bib25] Koolschijn P.C.M.P., Peper J.S., Crone E.A. (2014). The influence of sex steroids on structural brain maturation in adolescence. PLoS ONE.

[bib26] de Lange A.M.G., Anatürk M., Rokicki J., Han L.K.M., Franke K., Alnæs D., Ebmeier K.P., Draganski B., Kaufmann T., Westlye L.T., Hahn T., Cole J.H. (2022). Mind the gap: Performance metric evaluation in brain-age prediction. Hum. Brain Mapp..

[bib27] Lenroot R.K., Giedd J.N. (2010). Sex differences in the adolescent brain. Brain Cogn..

[bib28] Mattoni M., Wilson S., Olino T.M. (2021). Identifying profiles of brain structure and associations with current and future psychopathology in youth. Dev. Cogn. Neurosci..

[bib29] McLaughlin K.A., Weissman D., Bitrán D. (2019). Childhood adversity and neural development: a systematic review. Annu. Rev. Dev. Psychol..

[bib30] Mendle J. (2014). Why puberty matters for psychopathology. Child Dev. Perspect..

[bib31] Merz E.C., He X., Noble K.G. (2018). Anxiety, depression, impulsivity, and brain structure in children and adolescents. NeuroImage: Clin..

[bib32] Mills K.L., Goddings A.L., Herting M.M., Meuwese R., Blakemore S.J., Crone E.A., Dahl R.E., Güroğlu B., Raznahan A., Sowell E.R., Tamnes C.K. (2016). Structural brain development between childhood and adulthood: Convergence across four longitudinal samples. NeuroImage.

[bib33] Mueller S.C., Aouidad A., Gorodetsky E., Goldman D., Pine D.S., Ernst M. (2013). Gray matter volume in adolescent anxiety: An impact of the brain-derived neurotrophic factor Val66met polymorphism?. J. Am. Acad. Child Adolesc. Psychiatry.

[bib34] Neufang S., Specht K., Hausmann M., Güntürkün O., Herpertz-Dahlmann B., Fink G.R., Konrad K. (2009). Sex differences and the impact of steroid hormones on the developing human brain. Cereb. Cortex.

[bib35] Panizzon M.S., Fennema-Notestine C., Eyler L.T., Jernigan T.L., Prom-Wormley E., Neale M., Jacobson K., Lyons M.J., Grant M.D., Franz C.E., Xian H., Tsuang M., Fischl B., Seidman L., Dale A., Kremen W.S. (2009). Distinct genetic influences on cortical surface area and cortical thickness. Cereb. Cortex.

[bib36] Peper J.S., Brouwer R.M., Schnack H.G., van Baal G.C., van Leeuwen M., van den Berg S.M., Delemarre-Van de Waal H.A., Boomsma D.I., Kahn R.S., Hulshoff Pol H.E. (2009). Sex steroids and brain structure in pubertal boys and girls. Psychoneuroendocrinology.

[bib37] Peper J.S., Schnack H.G., Brouwer R.M., Van Baal G.C.M., Pjetri E., Székely E., Van Leeuwen M., Van Den Berg S.M., Collins D.L., Evans A.C., Boomsma D.I., Kahn R., Hulshoff Pol H.E. (2009). Heritability of regional and global brain structure at the onset of puberty: A magnetic resonance imaging study in 9-year-old twin pairs. Hum. Brain Mapp..

[bib38] Petersen A.C., Crockett L., Richards M., Boxer A. (1988). A self-report measure of pubertal status: Reliability, validity, and initial norms. J. Youth Adolesc..

[bib39] Rakesh D., Cropley V., Zalesky A., Vijayakumar N., Allen N.B., Whittle S. (2021). Neighborhood disadvantage and longitudinal brain-predicted-age trajectory during adolescence. Dev. Cogn. Neurosci..

[bib40] Rasmussen A.R., Wohlfahrt-Veje C., De Renzy-Martin K.T., Hagen C.P., Tinggaard J., Mouritsen A., Mieritz M.G., Main K.M. (2015). Validity of self-assessment of pubertal maturation. Pediatrics.

[bib41] Sawyer S.M., Azzopardi P.S., Wickremarathne D., Patton G.C. (2018). The age of adolescence. Lancet Child Adolesc. Health.

[bib42] Schmaal L., Veltman D.J., Van Erp T.G.M., Smann P.G., Frodl T., Jahanshad N., Loehrer E., Tiemeier H., Hofman A., Niessen W.J., Vernooij M.W., Ikram M.A., Wittfeld K., Grabe H.J., Block A., Hegenscheid K., Völzke H., Hoehn D., Czisch M., Hibar D.P. (2016). Subcortical brain alterations in major depressive disorder: Findings from the ENIGMA Major Depressive Disorder working group. Mol. Psychiatry.

[bib43] Schmaal L., Hibar D.P., Sämann P.G., Hall G.B., Baune B.T., Jahanshad N., Cheung J.W., Van Erp T.G.M., Bos D., Ikram M.A., Vernooij M.W., Niessen W.J., Tiemeier H., Hofman A., Wittfeld K., Grabe H.J., Janowitz D., Bülow R., Selonke M., Veltman D.J. (2017). Cortical abnormalities in adults and adolescents with major depression based on brain scans from 20 cohorts worldwide in the ENIGMA Major Depressive Disorder Working Group. Mol. Psychiatry.

[bib44] Schulz K.M., Molenda-Figueira H.A., Sisk C.L. (2009). Back to the future: the organizational-activational hypothesis adapted to puberty and adolescence. Horm. Behav..

[bib45] Sharma S., Arain Mathur, Rais Nel, Sandhu Haque, Johal (2013). Maturation of the adolescent brain. Neuropsychiatr. Dis. Treat..

[bib46] Shirtcliff E.A., Dahl R.E., Pollak S.D. (2009). Pubertal development: correspondence between hormonal and physical development. Child Dev..

[bib47] Sisk C.L., Foster D.L. (2004). The neural basis of puberty and adolescence. Nat. Neurosci..

[bib48] Dehestani, N., Vijayakumar, N., Ball, G., Mansour,L.,S, Whittle, S., Silk, T.J. (2023). *“Puberty a ge gap”: A new method of pubertal timing and its association with psychopathology*. 10.1101/2022.05.13.22275069. PMC11116096 38052980

[bib49] Tingley, D., Yamamoto, T., Hirose, K., Keele, L., Imai, K. (2014). Mediation: R package for casual mediation analysis.

[bib50] Ullsperger J.M., Nikolas M.A. (2017). A meta-analytic review of the association between pubertal timing and psychopathology in adolescence: Are there sex differences in risk?. Psychol. Bull..

[bib51] Urošević S., Collins P., Muetzel R., Lim K.O., Luciana M. (2014). Pubertal status associations with reward and threat sensitivities and subcortical brain volumes during adolescence. Brain Cogn..

[bib52] Vijayakumar N., Op de Macks Z., Shirtcliff E.A., Pfeifer J.H. (2018). Puberty and the human brain: Insights into adolescent development. Neurosci. Biobehav. Rev..

[bib53] Vijayakumar N., Youssef G.J., Allen N.B., Anderson V., Efron D., Hazell P., Mundy L., Nicholson J.M., Patton G., Seal M.L., Simmons J.G., Whittle S., Silk T. (2021). A longitudinal analysis of puberty‐related cortical development. NeuroImage.

[bib54] Wierenga L.M., Bos M.G.N., Schreuders E., vd Kamp F., Peper J.S., Tamnes C.K., Crone E.A. (2018). Unraveling age, puberty and testosterone effects on subcortical brain development across adolescence. Psychoneuroendocrinology.

