# Supplementary material for: Developmental brain changes during puberty and associations with mental health problems
Source: Dev Cogn Neurosci. 2023 Mar 9;60:101227. doi: 10.1016/j.dcn.2023.101227 (PMC10036507; doi:10.1016/j.dcn.2023.101227)
Supplement: Supplementary file 1 — Supplementary material [file mmc1.docx]

**Supplementary Information**

**Developmental brain changes during puberty and associations with psychopathology**

Niousha Dehestani^,1,2^, Sarah Whittle^2^, Nandita Vijayakumar^1*^ & Timothy J. Silk^1,3*^

^1^School of Psychology, Deakin University, 221 Burwood Highway, Burwood, VIC 3125, Australia.

^2^Melbourne Neuropsychiatry Centre, Department of Psychiatry, The University of Melbourne and Melbourne Health, Victoria, Australia

^3^Developmental Imaging, Murdoch Children’s Research Institute, Parkville 3052, Australia.

^*^ These authors jointly supervised this work: Nandita Vijayakumar and Timothy J. Silk

**Section 1. Cleaning and pre-processing data.**

To implement the brain age model, this study used the neuroimaging data from release 4.0 of ABCD study that was collected in baseline and two-year follow-up waves (N = 19,587 observations). This data contained structural measures extracted from T1-image information. Brain age gap was fitted using the measures of volume, thickness, and surface area across both longitudinal waves. We used the ABCD neuroimaging inclusion criteria which rated the T1 images as 0 (must exclude) and 1 (recommended for usage). We only kept individual sessions that were rated as 1. Therefore, a sample size of N = 19,097 observations (9,037 female) was available.

To implement the puberty age model, data was also used from release 4.0 of ABCD study including baseline, 2-year follow-up (N = 22,293 observations (10,642 female)). Participants who did not have both hormones and PDS, or family and site (N= 7094 Missing values) information were excluded from this study. To clean hormone data, we also excluded participants who did not match in biological sex (reported at the time of saliva collection) and their reported sex at birth. At the end, N= 15,199 observations (7301 female) remained. Additionally, we used Linear Mixed effect Models (LMM) to remove the confounding effect of using caffeine, having exercise, and time for collecting saliva sample that varied across the ABCD sample from 7 am to 7 pm. All the cleaning procedures for hormones were conducted as recommended by Herting et al., (2021).

This study only used participants who had both neuroimaging and pubertal information (N = 13,083 (6254) female)). Finally, to implement the brain age and puberty age model, we randomly selected a single time point from each participant to alleviate the potential bias created from using repeated measures in a cross-sectional design. This left a final sample size of N = 10,167 participants (4,894 female). We trained our brain and puberty age models on a typically developing sample (N =5,145 (2582 females)) defined by excluding participants who have scored >60 on any of the CBCL DSM-5 oriented scales including ADHD, conduct, Opposite, Somatic, Anxiety, Depression, Stress, and OCD, as well as internalising, externalising and total problems.

**Section 2. Choosing PCA component for brain age model**

In this study, we implemented a Principal Component Regression approach to increase the robustness of the machine learning model against noise and overfitting. To this end, the structural brain features were decomposed into orthogonal eigenmodes using a singular vector decomposition, also known as principal component analysis (PCA). We then sorted the components according to their respective ratio of total variance explained. We next selected the first N principal components such that the total selection explained more than 90% of the total variance (see Figure S1). This selection procedure ensured that the most important features captured by structural brain variations were included in the brain age model while the overall dimensionality was reduced. As a result of this procedure, a total of 90 principal components remained.


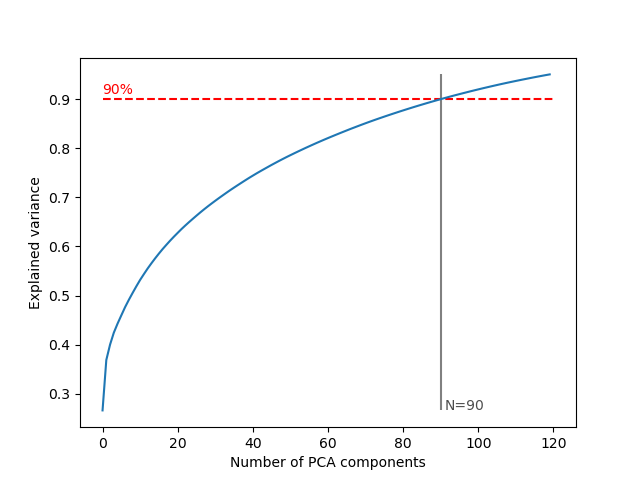


Figure S1. 90 components can capture 90% of variance.

**Section 3.  Accuracy of lobar brain age gap.**

The accuracy of the subcortical brain age model was r =0.34, MAE = 9.86 months, while the accuracy of the frontal brain age model was: r =0.36, mae = 9.85 months.

**Section 4.  Association between puberty age gap and brain age gap when controlling for BMI, race and SES.**

Additional mixed effects models investigated the association between puberty age gap and brain age gap covarying for BMI, race and SES as fixed effects. Results remained consistent to the main models without these covariates (see Table S1).

Table S1. Association between puberty age gap and brain age gap with BMI, race and SES as covariates.

|  | Female | | Male | |
| --- | --- | --- | --- | --- |
|  | T | *p* value | T | *p* value |
| Combined puberty age gap | **6.43** | **<0.001** | **2.96** | **<0.01** |
| Physical puberty age gap | 6.19 | <0.001 | 2.11 | <0.01 |
| Hormonal puberty age gap | 3.02 | <0.001 | 0.73 | 0.07 |

**Section 5.  Association between brain age gap and mental health problems longitudinally on ABCD study.**

We assessed the association between brain age gap and mental health problems in a longitudinal mediation model. Linear mixed effect model (LMM) was used to investigate the association between brain age gap at baseline and mental health problems at two-year follow-up, with age and mental health problems in the baseline as the fixed effect and site as the random effect, in males and females separately. Results showed that brain age gap was not significantly associated with mental health problems longitudinally. Each mental health outcome was examined in a separate linear mixed effects model, with a global brain age gap (Table S2). Additionally, we used the lobar brain age gap, including subcortical brain age gap in males and females (Table S3) and frontal brain age gap in females (Table S4), to investigate the association with mental health problems.

Table S2. Association between brain age gap in baseline and mental health problems in two-year follow-up.

|  | Total brain age | |
| --- | --- | --- |
|  | T | P |
| Total | 0.45 | 0.65 |
| Internalising | 0.52 | 0.60 |
| Externalising | -0.34 | 0.73 |
| Anxiety/Depression | 0.50 | 0.61 |
| Withdrawn/Depression | 2.04 | 0.04 |
| Somatic Complaints | 0.50 | 0.62 |
| Attention problems | 0.80 | 0.41 |
| Rule breaking | 1.05 | 0.29 |
| Aggressive Behaviour | 0.39 | 0.70 |
| Thought problems | 0.39 | 0.69 |
| Social problems | 0.08 | 0.94 |

Table S3. Association between subcortical brain age gap (in males and females) at baseline and mental health problems in two-year follow-up.

|  | Subcortical brain age | |
| --- | --- | --- |
|  | T | p |
| Total | -0.18 | 0.86 |
| Internalising | 0.97 | 0.33 |
| Externalising | -1.58 | 0.11 |
| Anxiety/Depression | 0.21 | 0.83 |
| Withdrawn/depression | -0.55 | 0.59 |
| Somatic Complaints | 1.55 | 0.12 |
| Attention Problems | -0.75 | 0.45 |
| Rule Breaking | -1.14 | 0.25 |
| Aggressive Behaviour | -1.17 | 0.24 |
| Thought Problems | -0.01 | 0.99 |
| Social Problems | -1.36 | 0.17 |

Table S4. Association between frontal brain age gap (in females) at baseline and mental health problems in two-year follow-up.

|  | Frontal brain age | |
| --- | --- | --- |
|  | T | **P** |
| Total | -0.51 | 0.60 |
| Internalising | -0.38 | 0.70 |
| Externalising | 0.69 | 0.48 |
| Anxiety/Depression | -1.43 | 0.15 |
| Withdrawn/Depression | 0.29 | 0.76 |
| Somatic Complaints | 0.65 | 0.51 |
| Attention problems | -0.77 | 0.43 |
| Rule breaking | 0.86 | 0.38 |
| Aggressive Behaviour | 0.68 | 0.49 |
| Thought problems | -0.59 | 0.55 |
| Social problems | 0.30 | 0.75 |

**Section 6. Mediation model between puberty age gap and mental health problems.**

We used a linear mixed-effects model to assess the mediating role of brain age gap in the association between puberty age gap and different mental health problems. Mediation analyses were conducted for subcortical brain age gap in males and both subcortical and frontal brain age gap in females. We also included age and baseline mental health as fixed effects and site as a random effect (see Table S5 and S6). Additionally, we repeated this analysis with the inclusion of BMI, race and SES as covariates that had similar result.

Table S5. Mediation role of subcortical brain age gap in association between puberty age gap at baseline and mental health problems in two-year follow-up.

|  | females | | | males | | |
| --- | --- | --- | --- | --- | --- | --- |
|  | ACME | ADE | Total Effect | ACME | ADE | Total Effect |
| Total | 0.73 | **<0.01** | **<0.01** | 0.31 | 0.09 | 0.10 |
| Internalising | 0.18 | **<0.01** | **<0.01** | 0.91 | 0.21 | 0.20 |
| Externalising | 0.22 | **<0.001** | **<0.001** | 0.09 | 0.15 | 0.19 |
| Anxiety/Depression | 0.56 | 0.61 | 0.06 | 0.53 | 0.38 | 0.42 |
| Withdrawn/Depression | 0.14 | **<0.001** | **<0.001** | 0.52 | 0.72 | 0.76 |
| Somatic Complaints | 0.24 | **<0.01** | **<0.01** | 0.57 | 0.08 | 0.08 |
| Attention problems | 0.86 | 0.55 | 0.56 | 0.25 | 0.54 | 0.57 |
| Rule breaking | 0.72 | **<0.001** | **<0.001** | 0.06 | 0.07 | 0.06 |
| Aggressive Behaviour | 0.30 | 0.03 | 0.03 | 0.33 | 0.26 | 0.27 |
| Thought problems | 0.29 | 0.38 | 0.33 | 0.32 | 0.25 | 0.29 |
| Social problems | 0.22 | **<0.001** | **<0.001** | 0.49 | 0.09 | 0.01 |

Table S6. Mediation role of frontal brain age gap in association between puberty age gap at baseline and mental health problems in two-year follow-up.

|  | Females | | |
| --- | --- | --- | --- |
|  | ACME | ADE | Total Effect |
| Total | 0.36 | **<0.001** | **<0.001** |
| Internalising | 0.62 | **<0.001** | **<0.001** |
| Externalising | 0.32 | **<0.001** | **<0.001** |
| Anxiety/Depression | 0.49 | 0.06 | 0.06 |
| Withdrawn/Depression | 0.99 | **<0.001** | **<0.001** |
| Somatic Complaints | 0.61 | **<0.001** | **<0.001** |
| Attention problems | 0.21 | 0.55 | 0.60 |
| Rule breaking | 0.45 | **<0.001** | **<0.001** |
| Aggressive Behaviour | 0.77 | **<0.01** | **<0.01** |
| Thought problems | 0.06 | 0.30 | 0.35 |
| Social problems | 0.13 | **<0.001** | **<0.001** |

**Section 7.  Implementing the brain age model using the HCP-D sample.** To ensure that training the brain age model on ABCD dataset with a relatively narrow age range did not potentially reduce power to predict mental health problems, we replicated the brain age analyses using Human Connectome Project Development (HCP-D) as a training sample for implementing the brain age model. HCP-D has participants aged 8 to 22 years old (N=652). T1-weighted images were used, again using FreeSurfer derived estimates of cortical thickness, volume and surface area (using the Desikan Killiany atlas) as well as subcortical volumes. Refer to the protocol paper (Harms et al., 2018) for more details on imaging acquisition and processing. These cortical and subcortical features were subsequently reduced by principal component analysis and the reduced feature set was used to train the model. We used support vector regression as our machine learning (ML) algorithm and performed 10-fold cross-validation to train the model on HCP-D data, followed by which the fitted model was tested on the ABCD data set (N = 10,167 (4894 female) the same as our main analysis for brain age). The accuracy of this brain age model was Mae = 1 year and 9-month, r = 0.86, p value < 0.001. Using this modified brain age model, we replicated those main findings of positive puberty age gap being related to positive brain age gap. Further, physical puberty age gap was the strongest predictor of brain maturation in both females and males. As with the main analyses, brain age gap was not associated with any dimensions of mental health problems longitudinally and did not mediate the relationship between puberty age gap and mental health problems.
